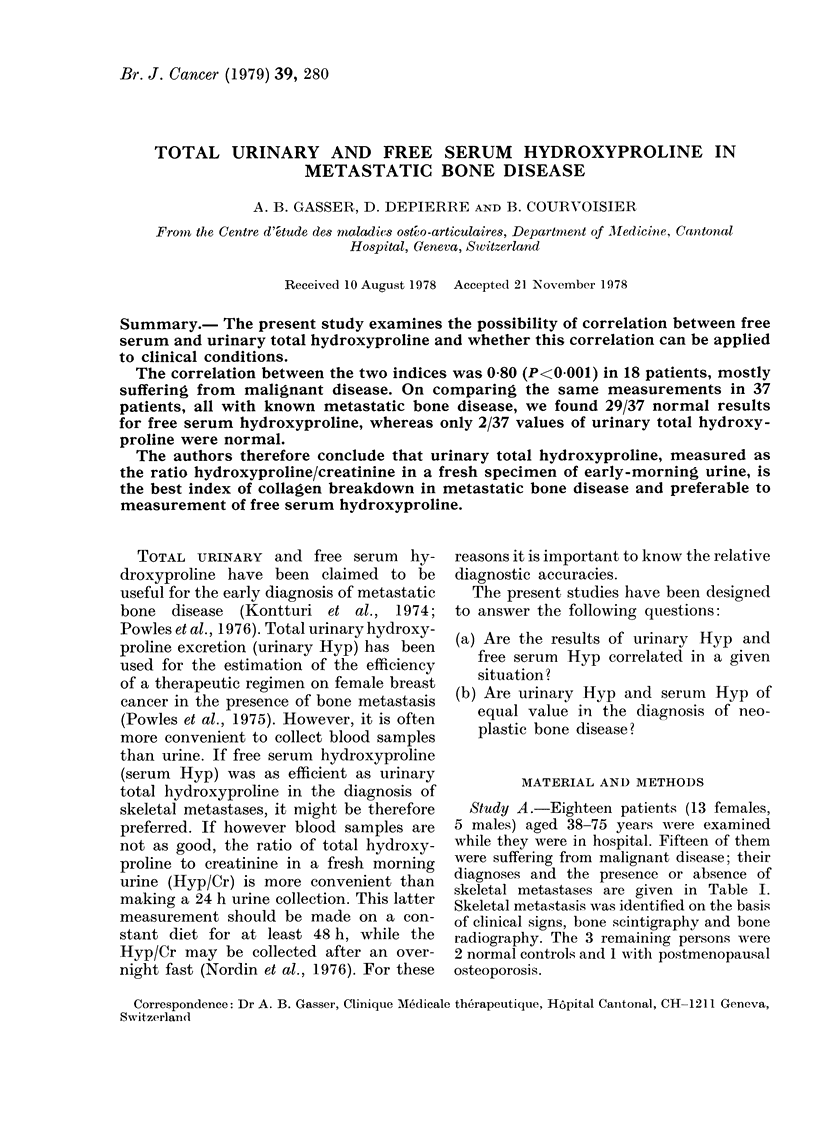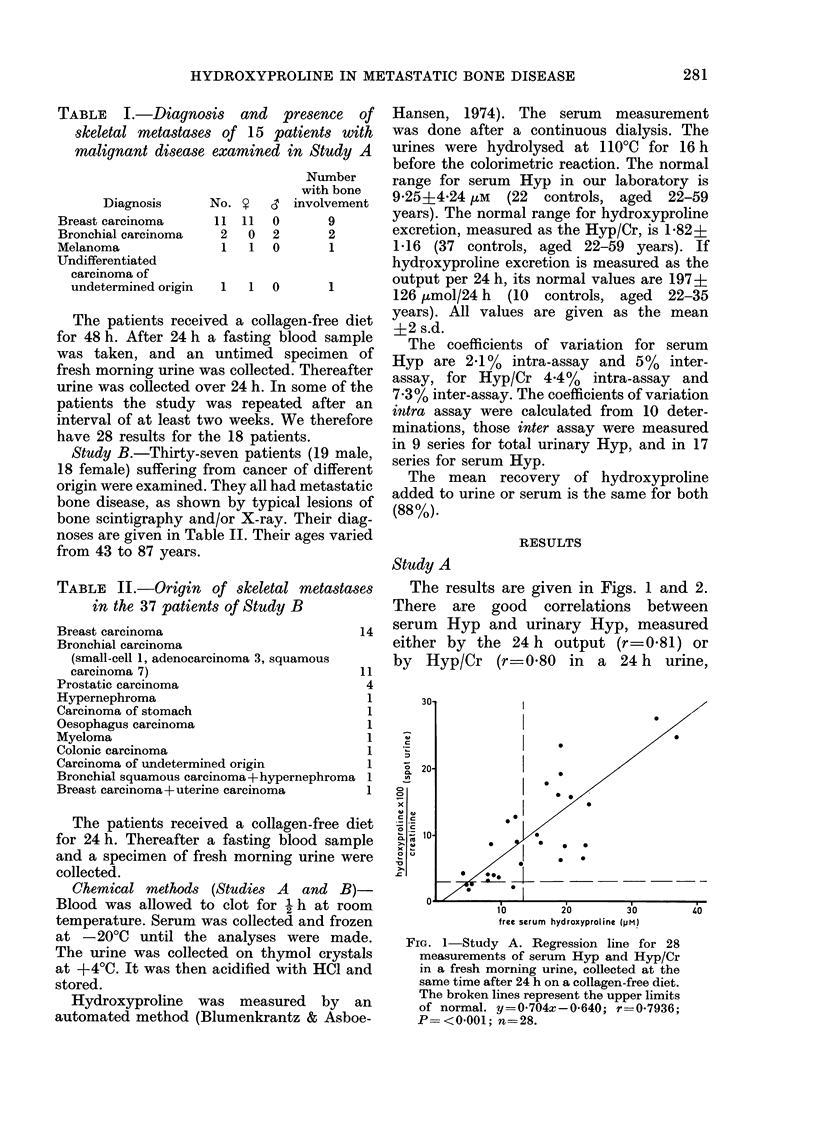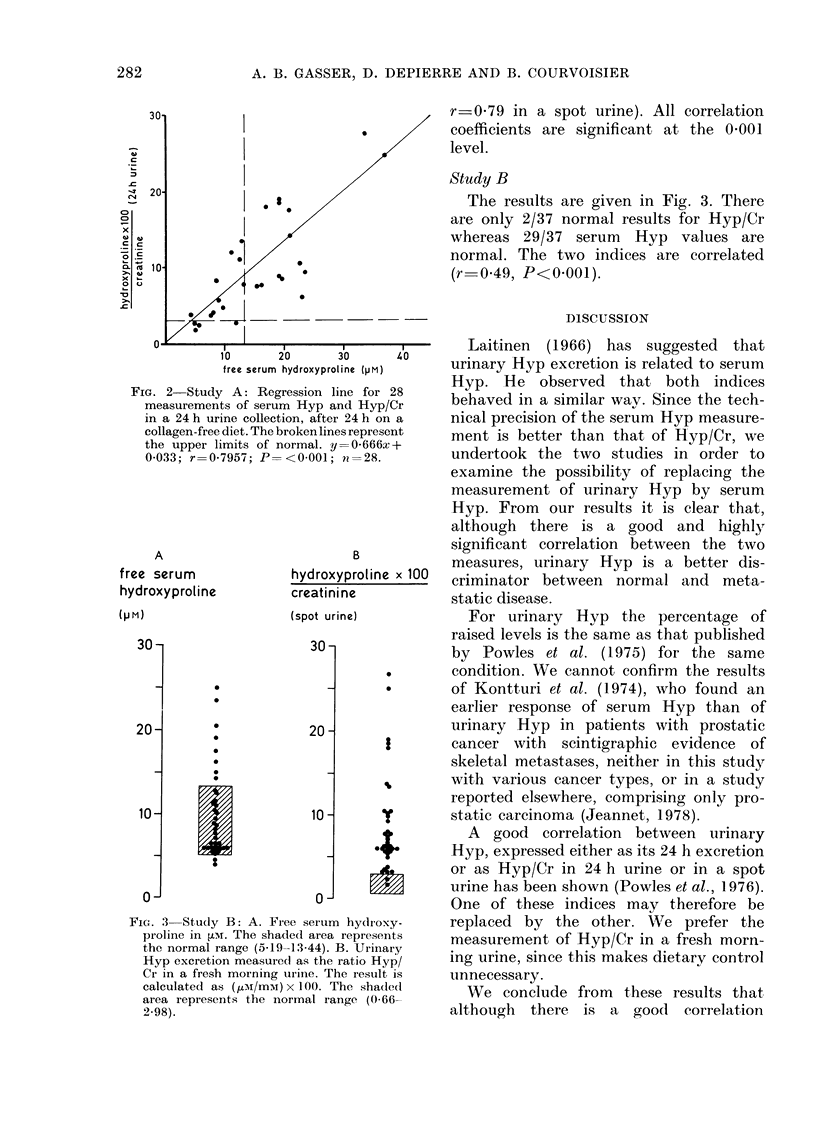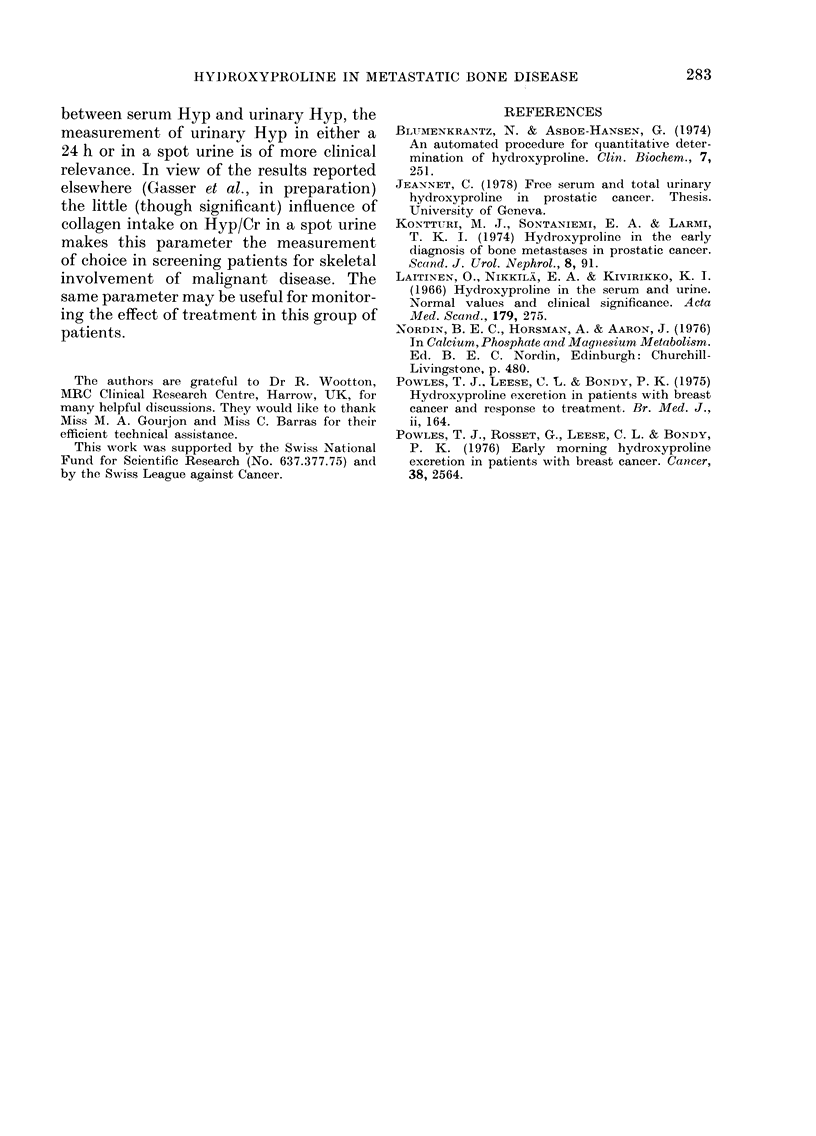# Total urinary and free serum hydroxyproline in metastatic bone disease.

**DOI:** 10.1038/bjc.1979.52

**Published:** 1979-03

**Authors:** A. B. Gasser, D. Depierre, B. Courvoisier

## Abstract

The present study examines the possibility of correlation between free serum and urinary total hydroxyproline and whether this correlation can be applied to clinical conditions. The correlation between the two indices was 0.80 (P less than 0.001) in 18 patients, mostly suffering from malignant disease. On comparing the same measurements in 37 patients, all with known metastatic bone disease, we found 29/37 normal results for free serum hydroxyproline, whereas only 2/37 values of urinary total hydroxyproline were normal. The authors therefore conclude that urinary total hydroxyproline, measured as the ratio hydroxyproline/creatinine in a fresh specimen of early-morning urine, is the best index of collagen breakdown in metastatic bone disease and preferable to measurement of free serum hydroxyproline.


*Br. J. Cancer* (1979) **39**, 280

### TOTAL URINARY AND FREE SERUM HYDROXYPROLINE IN METASTATIC BONE DISEASE

A. B. GASSER, D. DEPIERRE AND B. COURVOISIER

*From the Centre d'étude des maladies ostéo-articulaires, Department of Medicine, Cantonal Hospital, Geneva, Switzerland*

Received 10 August 1978 Accepted 21 November 1978

**Summary.— The present study examines the possibility of correlation between free serum and urinary total hydroxyproline and whether this correlation can be applied to clinical conditions.**

**The correlation between the two indices was 0·80 ($P<0·001$) in 18 patients, mostly suffering from malignant disease. On comparing the same measurements in 37 patients, all with known metastatic bone disease, we found 29/37 normal results for free serum hydroxyproline, whereas only 2/37 values of urinary total hydroxyproline were normal.**

**The authors therefore conclude that urinary total hydroxyproline, measured as the ratio hydroxyproline/creatinine in a fresh specimen of early-morning urine, is the best index of collagen breakdown in metastatic bone disease and preferable to measurement of free serum hydroxyproline.**

TOTAL URINARY and free serum hydroxyproline have been claimed to be useful for the early diagnosis of metastatic bone disease (Kontturi *et al.*, 1974; Powles *et al.*, 1976). Total urinary hydroxyproline excretion (urinary Hyp) has been used for the estimation of the efficiency of a therapeutic regimen on female breast cancer in the presence of bone metastasis (Powles *et al.*, 1975). However, it is often more convenient to collect blood samples than urine. If free serum hydroxyproline (serum Hyp) was as efficient as urinary total hydroxyproline in the diagnosis of skeletal metastases, it might be therefore preferred. If however blood samples are not as good, the ratio of total hydroxyproline to creatinine in a fresh morning urine (Hyp/Cr) is more convenient than making a 24 h urine collection. This latter measurement should be made on a constant diet for at least 48 h, while the Hyp/Cr may be collected after an overnight fast (Nordin *et al.*, 1976). For these reasons it is important to know the relative diagnostic accuracies.

The present studies have been designed to answer the following questions:

(a) Are the results of urinary Hyp and free serum Hyp correlated in a given situation?

(b) Are urinary Hyp and serum Hyp of equal value in the diagnosis of neoplastic bone disease?

### MATERIAL AND METHODS

*Study A*.—Eighteen patients (13 females, 5 males) aged 38–75 years were examined while they were in hospital. Fifteen of them were suffering from malignant disease; their diagnoses and the presence or absence of skeletal metastases are given in Table I. Skeletal metastasis was identified on the basis of clinical signs, bone scintigraphy and bone radiography. The 3 remaining persons were 2 normal controls and 1 with postmenopausal osteoporosis.

Correspondence: Dr A. B. Gasser, Clinique Médicale thérapeutique, Hôpital Cantonal, CH–1211 Geneva, Switzerland

TABLE I.—*Diagnosis and presence of skeletal metastases of* 15 *patients with malignant disease examined in Study A*

| Diagnosis | No. | ♀ | ♂ | Number with bone involvement |
|---|---|---|---|---|
| Breast carcinoma | 11 | 11 | 0 | 9 |
| Bronchial carcinoma | 2 | 0 | 2 | 2 |
| Melanoma | 1 | 1 | 0 | 1 |
| Undifferentiated carcinoma of undetermined origin | 1 | 1 | 0 | 1 |

The patients received a collagen-free diet for 48 h. After 24 h a fasting blood sample was taken, and an untimed specimen of fresh morning urine was collected. Thereafter urine was collected over 24 h. In some of the patients the study was repeated after an interval of at least two weeks. We therefore have 28 results for the 18 patients.

*Study B*.—Thirty-seven patients (19 male, 18 female) suffering from cancer of different origin were examined. They all had metastatic bone disease, as shown by typical lesions of bone scintigraphy and/or X-ray. Their diagnoses are given in Table II. Their ages varied from 43 to 87 years.

TABLE II.—*Origin of skeletal metastases in the* 37 *patients of Study B*

| | |
|---|---|
| Breast carcinoma | 14 |
| Bronchial carcinoma (small-cell 1, adenocarcinoma 3, squamous carcinoma 7) | 11 |
| Prostatic carcinoma | 4 |
| Hypernephroma | 1 |
| Carcinoma of stomach | 1 |
| Oesophagus carcinoma | 1 |
| Myeloma | 1 |
| Colonic carcinoma | 1 |
| Carcinoma of undetermined origin | 1 |
| Bronchial squamous carcinoma + hypernephroma | 1 |
| Breast carcinoma + uterine carcinoma | 1 |

The patients received a collagen-free diet for 24 h. Thereafter a fasting blood sample and a specimen of fresh morning urine were collected.

*Chemical methods* (*Studies A and B*)—Blood was allowed to clot for ½ h at room temperature. Serum was collected and frozen at −20°C until the analyses were made. The urine was collected on thymol crystals at +4°C. It was then acidified with HCl and stored.

Hydroxyproline was measured by an automated method (Blumenkrantz & Asboe-Hansen, 1974). The serum measurement was done after a continuous dialysis. The urines were hydrolysed at 110°C for 16 h before the colorimetric reaction. The normal range for serum Hyp in our laboratory is 9·25±4·24 $\mu$M (22 controls, aged 22–59 years). The normal range for hydroxyproline excretion, measured as the Hyp/Cr, is 1·82± 1·16 (37 controls, aged 22–59 years). If hydroxyproline excretion is measured as the output per 24 h, its normal values are 197± 126 $\mu$mol/24 h (10 controls, aged 22–35 years). All values are given as the mean ±2 s.d.

The coefficients of variation for serum Hyp are 2·1% intra-assay and 5% inter-assay, for Hyp/Cr 4·4% intra-assay and 7·3% inter-assay. The coefficients of variation *intra* assay were calculated from 10 determinations, those *inter* assay were measured in 9 series for total urinary Hyp, and in 17 series for serum Hyp.

The mean recovery of hydroxyproline added to urine or serum is the same for both (88%).

### RESULTS

### *Study A*

The results are given in Figs. 1 and 2. There are good correlations between serum Hyp and urinary Hyp, measured either by the 24 h output ($r$=0·81) or by Hyp/Cr ($r$=0·80 in a 24 h urine,

FIG. 1—Study A. Regression line for 28 measurements of serum Hyp and Hyp/Cr in a fresh morning urine, collected at the same time after 24 h on a collagen-free diet. The broken lines represent the upper limits of normal. $y$=0·704$x$−0·640; $r$=0·7936; $P$=<0·001; $n$=28.

FIG. 2—Study A: Regression line for 28 measurements of serum Hyp and Hyp/Cr in a 24 h urine collection, after 24 h on a collagen-free diet. The broken lines represent the upper limits of normal. $y=0{\cdot}666x+0{\cdot}033$; $r=0{\cdot}7957$; $P=<0{\cdot}001$; $n=28$.

FIG. 3—Study B: A. Free serum hydroxyproline in μM. The shaded area represents the normal range (5·19–13·44). B. Urinary Hyp excretion measured as the ratio Hyp/Cr in a fresh morning urine. The result is calculated as (μM/mM) × 100. The shaded area represents the normal range (0·66–2·98).

$r=0{\cdot}79$ in a spot urine). All correlation coefficients are significant at the 0·001 level.

*Study B*

The results are given in Fig. 3. There are only 2/37 normal results for Hyp/Cr whereas 29/37 serum Hyp values are normal. The two indices are correlated ($r=0{\cdot}49$, $P<0{\cdot}001$).

### DISCUSSION

Laitinen (1966) has suggested that urinary Hyp excretion is related to serum Hyp. He observed that both indices behaved in a similar way. Since the technical precision of the serum Hyp measurement is better than that of Hyp/Cr, we undertook the two studies in order to examine the possibility of replacing the measurement of urinary Hyp by serum Hyp. From our results it is clear that, although there is a good and highly significant correlation between the two measures, urinary Hyp is a better discriminator between normal and metastatic disease.

For urinary Hyp the percentage of raised levels is the same as that published by Powles *et al.* (1975) for the same condition. We cannot confirm the results of Kontturi *et al.* (1974), who found an earlier response of serum Hyp than of urinary Hyp in patients with prostatic cancer with scintigraphic evidence of skeletal metastases, neither in this study with various cancer types, or in a study reported elsewhere, comprising only prostatic carcinoma (Jeannet, 1978).

A good correlation between urinary Hyp, expressed either as its 24 h excretion or as Hyp/Cr in 24 h urine or in a spot urine has been shown (Powles *et al.*, 1976). One of these indices may therefore be replaced by the other. We prefer the measurement of Hyp/Cr in a fresh morning urine, since this makes dietary control unnecessary.

We conclude from these results that although there is a good correlation between serum Hyp and urinary Hyp, the measurement of urinary Hyp in either a 24 h or in a spot urine is of more clinical relevance. In view of the results reported elsewhere (Gasser *et al.*, in preparation) the little (though significant) influence of collagen intake on Hyp/Cr in a spot urine makes this parameter the measurement of choice in screening patients for skeletal involvement of malignant disease. The same parameter may be useful for monitoring the effect of treatment in this group of patients.

The authors are grateful to Dr R. Wootton, MRC Clinical Research Centre, Harrow, UK, for many helpful discussions. They would like to thank Miss M. A. Gourjon and Miss C. Barras for their efficient technical assistance.

This work was supported by the Swiss National Fund for Scientific Research (No. 637.377.75) and by the Swiss League against Cancer.

### REFERENCES

BLUMENKRANTZ, N. & ASBOE-HANSEN, G. (1974) An automated procedure for quantitative determination of hydroxyproline. *Clin. Biochem.*, **7**, 251.

JEANNET, C. (1978) Free serum and total urinary hydroxyproline in prostatic cancer. Thesis. University of Geneva.

KONTTURI, M. J., SONTANIEMI, E. A. & LARMI, T. K. I. (1974) Hydroxyproline in the early diagnosis of bone metastases in prostatic cancer. *Scand. J. Urol. Nephrol.*, **8**, 91.

LAITINEN, O., NIKKILÄ, E. A. & KIVIRIKKO, K. I. (1966) Hydroxyproline in the serum and urine. Normal values and clinical significance. *Acta Med. Scand.*, **179**, 275.

NORDIN, B. E. C., HORSMAN, A. & AARON, J. (1976) In *Calcium, Phosphate and Magnesium Metabolism.* Ed. B. E. C. Nordin, Edinburgh: Churchill-Livingstone, p. 480.

POWLES, T. J., LEESE, C. L. & BONDY, P. K. (1975) Hydroxyproline excretion in patients with breast cancer and response to treatment. *Br. Med. J.*, ii, 164.

POWLES, T. J., ROSSET, G., LEESE, C. L. & BONDY, P. K. (1976) Early morning hydroxyproline excretion in patients with breast cancer. *Cancer*, **38**, 2564.